# Effects of elexacaftor/tezacaftor/ivacaftor therapy on mental health of patients with cystic fibrosis

**DOI:** 10.3389/fphar.2023.1179208

**Published:** 2023-04-21

**Authors:** Linus Piehler, Ralf Thalemann, Christine Lehmann, Stephanie Thee, Jobst Röhmel, Zulfiya Syunyaeva, Mirjam Stahl, Marcus A. Mall, Simon Y. Graeber

**Affiliations:** ^1^ Department of Pediatric Respiratory Medicine, Immunology and Critical Care Medicine and Cystic Fibrosis Center, Charité - Universitätsmedizin Berlin, Corporate member of Freie Universität Berlin and Humboldt-Universität zu Berlin, Berlin, Germany; ^2^ German Center for Lung Research (DZL), Associated partner site, Berlin, Germany; ^3^ Berlin Institute of Health at Charité, Charité—Universitätsmedizin, Berlin, Germany

**Keywords:** cystic fibrosis, elexacaftor/tezacaftor/ivacaftor, mental health, depression, anxiety

## Abstract

**Introduction:** The CFTR modulator drug elexacaftor/tezacaftor/ivacaftor (ETI) was shown to improve CFTR function and clinical symptoms in patients with cystic fibrosis (CF) with at least one *F508del* allele. Recently, some case reports suggested potential side effects of ETI on mental health with an increase in depressive symptoms and even suicide attempts in patients with CF. However, the general effects of this triple combination therapy on the mental health status of patients with CF remain largely unknown.

**Methods:** We, therefore, performed a prospective, observational study in a real-life setting and investigated the relationship between initiation of ETI therapy and changes in mental health in adult patients with CF. We assessed Cystic Fibrosis Questionnaire-Revised (CFQ-R), Patient Health Questionnaire-9 (PHQ-9), Beck’s Depression Inventory – Fast Screen (BDI-FS) and Generalized Anxiety Disorder 7-item Scale (GAD-7) at baseline and 8–16 weeks after initiation of ETI.

**Results:** In total, 70 adult patients with CF with at least one *F508del* allele and a median age of 27.9 years were recruited. After initiation of ETI, the CFQ-R respiratory domain score improved by 27.9 (IQR 5.6 to 47.2; *p* < 0.001). The PHQ-9 score of depressive symptoms decreased by 1.0 (IQR -3.0 to 0.3; *p* < 0.05) with an increase of 16.9% in the group with a minimal score after initiation of ETI and a decrease in the groups of mild (−11.3%) or moderate (−5.7%) scores compared to baseline. The BDI-FS score of depressive symptoms decreased from 1.0 (IQR 0.0–2.0) at baseline to 0.0 (IQR 0.0 to 2.0; *p* < 0.05) after initiation of ETI. The group with a minimal BDI-FS score increased by 8.0% after initiation of ETI, whereas the groups with mild (−4.9%), moderate (−1.6%) or severe (−1.6%) scores decreased compared to baseline. The GAD-7 score of anxiety symptoms did not change after initiation of ETI compared to baseline (0.0; IQR -2.0. to 0.0; *p* = 0.112).

**Conclusion:** Initiation of ETI improves symptoms of depression in adult patients with CF with at least one *F508del* allele. However, symptoms of anxiety do not change after short-term therapy with ETI.

## 1 Introduction

Cystic fibrosis (CF) is an autosomal recessive disorder and the most common fatal monogenetic disease in Caucasian populations (Bell et al., 2020). Mutations in the cystic fibrosis transmembrane conductance regulator (*CFTR*) gene cause impaired chloride and bicarbonate transport in epithelial organs leading to a multi-organ disease affecting mainly the lungs, gastrointestinal tract and the pancreas (Bell et al., 2020; Mall et al., 2020). In adult patients with CF, symptoms of depression are observed in ∼20% and symptoms of anxiety in ∼30%, which is about 2-fold higher than in the general population (∼10% and ∼15%, respectively) (Martin et al., 2006; Goldbeck et al., 2010; Besier and Goldbeck, 2011; Ploessl et al., 2014; Quittner et al., 2014; Graziano et al., 2020; Terlizzi and Villarroel, 2020). Symptoms of depression and anxiety are associated with reduced quality of life and adherence to airway clearance treatment (Riekert et al., 2007; Smith et al., 2010; Yohannes et al., 2012), as well as disease progression including decline in lung function, an increased rate of pulmonary exacerbations and increased mortality in patients with CF (Riekert et al., 2007; Fidika et al., 2014; Schechter et al., 2021).

Recently, it was shown that the CFTR modulator triple combination therapy with elexacaftor, tezacaftor and ivacaftor (ETI) leads to unprecedented improvements in lung function, body mass index (BMI) and self-reported respiratory symptoms in clinical trials and real-world studies in CF patients with at least one *F508del* allele (Heijerman et al., 2019; Middleton et al., 2019; Barry et al., 2021; Burgel et al., 2021; Griese et al., 2021; Graeber et al., 2022a; Nichols et al., 2022). Further, we recently showed that ETI improves F508del-CFTR function to levels of 40%–50% of normal CFTR activity in the airways and intestine, and increases lung ventilation and improves mucus plugging and other morphological changes in the lungs of patients with CF with one or two *F508del* alleles (Graeber et al., 2022a; Graeber et al., 2022b). Besides these beneficial effects, some case reports describe increased symptoms of depression and anxiety in patients with CF starting with ETI therapy (Tindell et al., 2020; Ladores and Polen, 2021; Heo et al., 2022; Arslan et al., 2023). However, the effects of ETI on depression and anxiety have not been prospectively assessed in patients with CF.

The aim of this study was, therefore, to assess the effect of ETI on depression and anxiety in adult patients with CF. To achieve this goal, we performed a prospective, observational study in 70 patients with CF and one or two *F508del* alleles and investigated quality of life with the Cystic Fibrosis Questionnaire-Revised (CFQ-R), symptoms of depression with the Patient Health Questionnaire-9 (PHQ-9) and the Beck’s Depression Inventory - Fast Screen (BDI-FS) as well as symptoms of anxiety with the Generalized Anxiety Disorder 7-item Scale (GAD-7) at baseline and 8–16 weeks after initiation of ETI therapy.

## 2 Methods

### 2.1 Study population

This prospective observational post-approval study was conducted at the Christiane Herzog CF Center at Charité - Universitätsmedizin Berlin. The study was approved by the ethics committee of the Charité - Universitätsmedizin Berlin (EA2/220/18) and written informed consent was obtained from all patients included in the study. Patients were eligible to participate if they were at least 18 years old, diagnosed with CF and at least one *F508del* mutation, had no prior exposure to ETI and were willing to remain on a stable medication regimen including ETI according to the patient labeling and the prescribing information for the duration of study participation.

**FIGURE 1:**
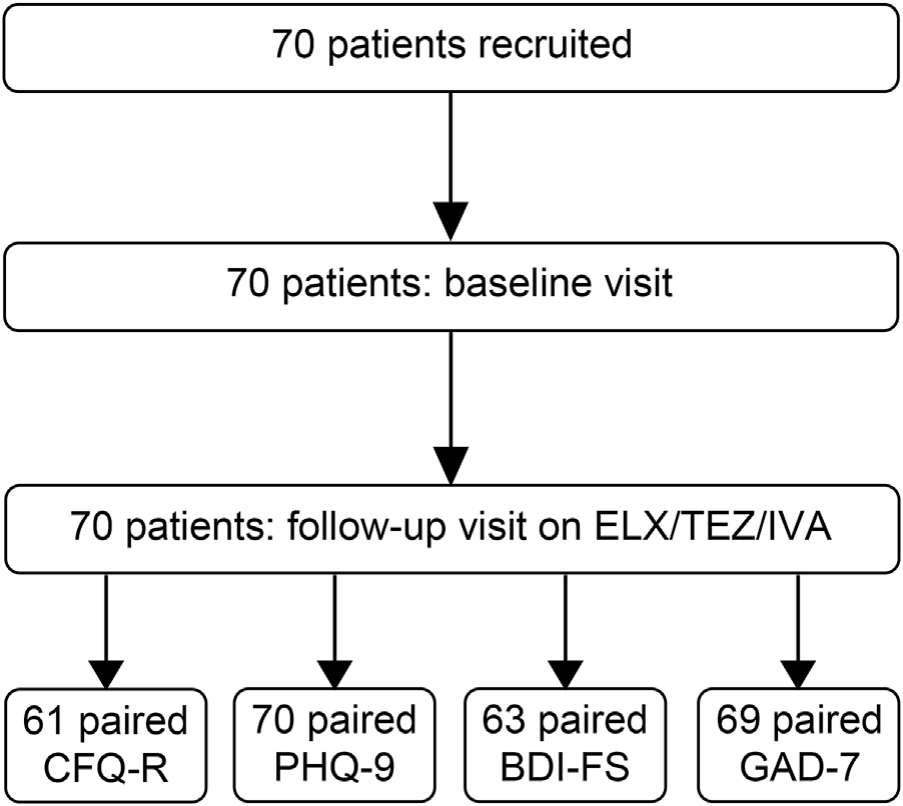
Flow chart of recruited patients with cystic fibrosis (CF) with at least one *F508del* mutation. BDI-FS = Beck’s Depression Inventory-FastScreen, CFQ-R = Cystic Fibrosis Questionnaire-Revised, ETI = elexacaftor/tezacaftor/ivacaftor, GAD-7 = Generalized Anxiety Disorder Scale-7 Items, PHQ-9 = Patient Health Questionnaire-9.

CFQ-R, PHQ-9, BDI-FS and GAD-7 scores were assessed at baseline and 8–16 weeks after initiation of therapy with the approved dose of ELX 200 mg and TEZ 100 mg every 24 h in combination with IVA 150 mg every 12 h (Figure 1).

### 2.2 Mental health screening

To determine the effect of ETI on quality of life in patients with CF, we assessed the CFQ-R at baseline and after initiation of therapy. The CFQ-R is a questionnaire validated in CF patients to record the health-related quality of life. The questionnaire contains a total of 50 items, which in turn are divided into 12 different domains (physical functioning, emotional functioning, social functioning/school functioning, body image, eating problems, treatment burden, respiratory symptoms, digestive symptoms, vitality, health perceptions, weight, role functioning). Each domain has a range from 0 to 100, with higher scores indicating a higher patient-reported quality of life (Quittner et al., 2005; Quittner et al., 2012).

To determine the effect of ETI on symptoms of depression in patients with CF, we assessed the PHQ-9 and BDI-FS questionnaire at baseline and after initiation of therapy. The PHQ-9 is a questionnaire for the detection of depressive symptoms (Kroenke et al., 2001). It identifies depressive symptoms present within the last 2 weeks. Scores ranging from 0 to 4 are considered to be minimal depressive values, scores from 5 to 9 indicate mild depression, scores from 10 to 14 moderate depression and scores ≥15 indicate severe depression. The maximum score is 27. The cut-off value for clinically relevant depressive symptoms was set at ≥10.

Since the PHQ-9 contains several items, whose variability may also be influenced by exacerbations and/or the course of CF (e.g., lack of energy, sleep disorders, loss of appetite), we used the BDI-FS as a second validated instrument to assess depression without somatic criteria. The BDI-FS is intended for use in clinical cohorts with severe underlying somatic illness (Poole et al., 2009) and measures the severity of depression by assessing non-somatic criteria for the diagnosis of major depressive disorder according to Diagnostic and Statistical Manual of Mental Disorders (DSM-IV) and (DSM-V) (Kliem et al., 2014). Scores ranging from 0 to 3 are considered to be minimal depressive values, scores from 4 to 6 indicate mild depression, scores from 7 to 9 moderate depression and scores ≥10 indicate severe depression. The maximum score is 21.

To determine the effect of ETI on symptoms of anxiety in patients with CF, we assessed the GAD-7 questionnaire at baseline and after initiation of therapy. The GAD-7 is a questionnaire for recording anxiety symptoms (Spitzer et al., 2006). It assesses anxiety-related complaints in the last 2 weeks. Scores ranging from 0 to 4 are minimal anxiety values, scores between 5 and 9 indicate mild generalized anxiety, scores from 10 to 14 describe moderate anxiety and scores ≥15 indicate severe generalized anxiety. The maximum score is 21. The cut-off value for clinically relevant anxiety symptoms was set at ≥10.

### 2.3 Statistical analysis

All data were analyzed with GraphPad Prism version 9.0.1 (GraphPad Software, San Diego, CA, USA) and R 3.6.2 (R Core Team, 2018). The data were not normally distributed and are presented as median and interquartile range (IQR). Comparisons between baseline and follow-up were tested by Wilcoxon signed-rank test. Subgroup analysis were performed in male and female patients. *p* < 0.05 was accepted to indicate statistical significance.

## 3 Results

### 3.1 Characteristics of study population

**FIGURE 2:**
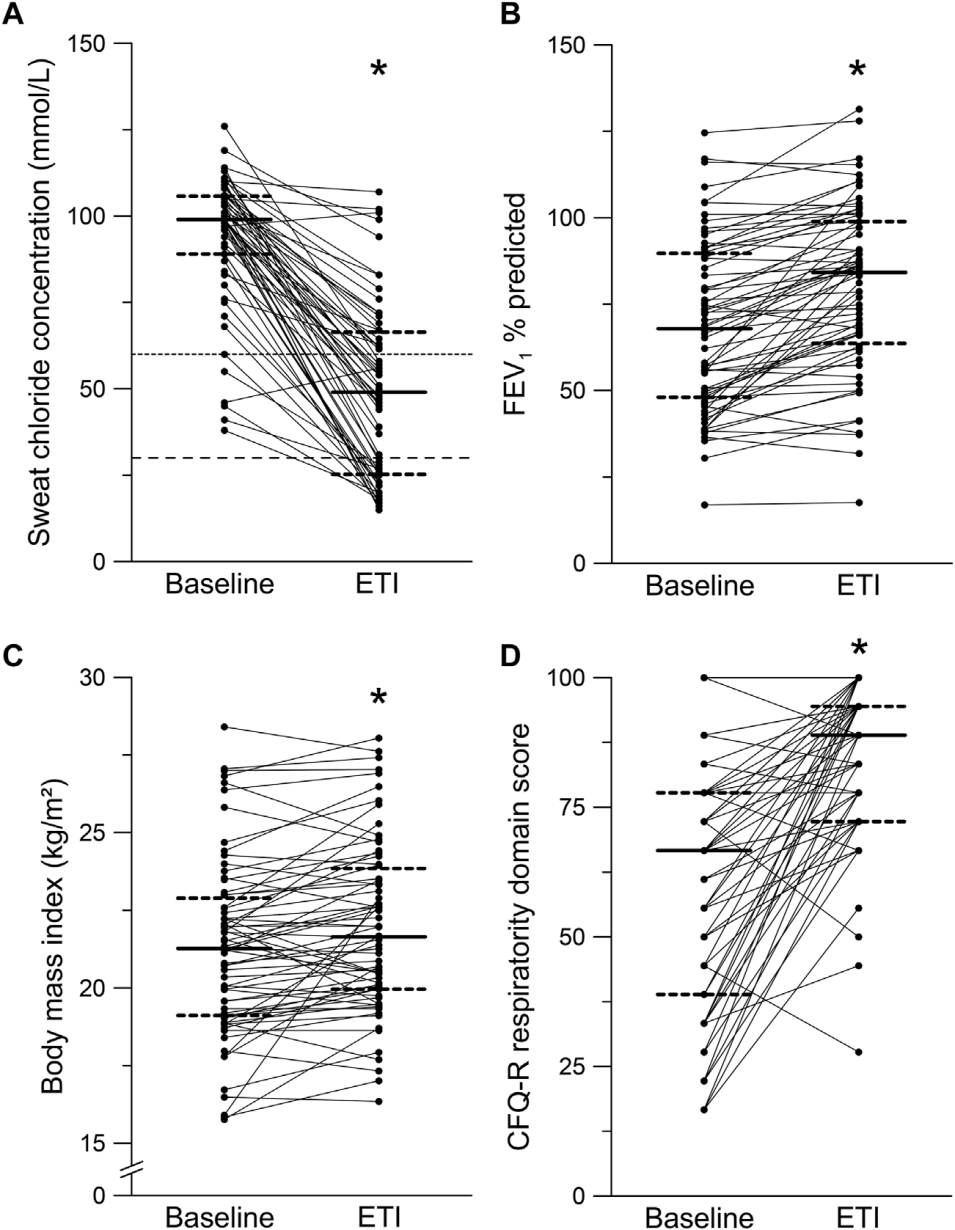
Effects of elexacaftor/tezacaftor/ivacaftor (ETI) on sweat chloride concentration, percent predicted forced expiratory volume in one second (FEV1% predicted), body mass index (BMI) and Cystic Fibrosis Questionnaire-Revised (CFQ-R) - respiratory domain. **(A–D)** Paired measurements of sweat chloride concentration **(A)**, FEV1% predicted **(B)**, BMI **(C)** and CFQ-R respiratory domain score in patients with CF and at least one *F508del* mutation at baseline and after initiation of ETI therapy. Solid lines represent the group median and dashed lines represent 25th and 75th percentile. *p < 0.001 compared with baseline.

In total, 70 adult patients with CF were enrolled between September 2020 and August 2021 to assess quality of life, symptoms of depression and anxiety as well as anthropometry, spirometry, and sweat chloride concentration at baseline and 8–16 weeks after initiation of ETI therapy (Figure 1). The median age of patients at baseline was 27.9 years (IQR 22.5 – 34.1) and 51.4% were female (Table 1). 45.7% of the patients were *F508del* homozygous and the other patients were heterozygous for *F508del* and a minimal function mutation (31.4%), a residual function mutation (21.4%) or a not identified mutation (1.4%). At baseline, 64.3% of the patients had not been on previous CFTR modulator therapy, 18.6% were on treatment with tezacaftor/ivacaftor, 14.3% were on treatment with lumacaftor/ivacaftor, and 2.9% were on treatment with ivacaftor (Table 1). Patients had a median forced expiratory volume in one second % predicted (ppFEV1) of 67.3% (IQR 48.0–88.7) and BMI of 21.3 kg/m^2^ (IQR 19.1–23.0) (Table 1). In our cohort, sweat chloride concentration decreased by 44.5 mmol/L (IQR -63.5 to −28.5; *p* < 0.001; Figure 2A), ppFEV1 improved by 12.1% (IQR 2.5 – 18.0; *p* < 0.001; Figure 2B), and BMI increased by 0.5 kg/m^2^ (IQR -0.2 to 1.2; *p* < 0.001; Figure 2C) after initiation of ETI.

**TABLE 1:** Clinical characteristics of patients with cystic fibrosis at baseline.

Patient characteristics at baseline	Median (IQR) or n (%)
Patient sample size	70
Age (years)	27.9 (22.5–34.1)
Female sex at birth	36 (51.4%)
*CFTR* genotype	
*F508del*/*F508del*	32 (45.7%)
*F508del*/minimal function mutation	22 (31.4%)
*F508del*/residual function mutation	15 (21.4%)
*F508del*/mutation not identified	1 (1.4%)
CFTR modulator therapy at baseline	
none	45 (64.3%)
Ivacaftor	2 (2.9%)
Lumacaftor/Ivacaftor	10 (14.3%)
Tezacaftor/Ivacaftor	13 (18.6%)
Pancreatic insufficiency	59 (84.3%)
CF-related diabetes	17 (24.3%)
CF-related liver disease	17 (24.3%)
CF-related arthropathy	13 (18.6%)
FEV_1_% predicted	67.3 (48.0–88.4)
BMI (kg/m^2^)	21.3 (19.1–23.0)

BMI, body mass index; *CFTR*, cystic fibrosis transmembrane conductance regulator, FEV1% predicted = percent predicted forced expiratory volume in one second.

61 (87%) patients completed the CFQ-R, 70 patients (100%) completed the PHQ-9 questionnaire, 69 patients (99%) completed the GAD-7 questionnaire and 63 patients (90%) completed the BDI-FS questionnaire at baseline and after initiation of ETI (Figure 1).

### 3.2 Quality of life

The respiratory domain score of the CFQ-R, assessing the respiratory symptoms and previously used in clinical trials, increased by 27.9 (IQR 5.6 – 47.2; *p* < 0.001) (Figure 2D; Table 2). In addition, the domain scores for physical functioning (*p* < 0.001), social functioning/school functioning (*p* < 0.01), body image (*p* < 0.001), treatment burden (*p* < 0.001), vitality (*p* < 0.001), health perceptions (*p* < 0.001), and role functioning (*p* < 0.001) were improved after initiation of ETI (Table 2). On the other hand, no changes after initiation of ETI were observed for the domains emotional functioning (*p* = 0.372), eating problems (*p* = 0.319), digestive symptoms (*p* = 0.860) and weight (*p* = 0.825) (Table 2). A subgroup analysis according to gender revealed that the CFQ-R respiratory domain score improved in female 22.2 (IQR 11.1 to 38.9, *p* < 0.001) as well as male patients with CF 22.2 (IQR 6.9 to 52.8, *p* < 0.001) (Table 3).

**TABLE 2:** Effects of elexacaftor/tezacaftor/ivacaftor (ETI) on quality of life determined by the Cystic Fibrosis Questionnaire-Revised (CFQ-R).

				
CFQ-R domain	Baseline median (IQR)	ETI median (IQR)	Change between baseline and ETI median (IQR)	*p*-Value
Physical functioning	70.8 (45.8–89.6)	89.6 (75.0–100.0)	12.5 (4.2–29.2)	<0.001
Emotional functioning	80.0 (66.7–90.0)	80.0 (66.7–93.3)	0.0 (−6.7 to 6.7)	0.372
Social functioning/school functioning	66.7 (44.4–83.3)	72.2 (50.0–83.3)	5.6 (−5.6–16.7)	<0.01
Body image	66.7 (50.0–88.9)	77.8 (55.6–100.0)	11.1 (0.0–11.1)	<0.001
Eating problems	100.0 (77.8–100.0)	100.0 (88.9–100.0)	0.0 (0.0–0.0)	0.319
Treatment burden	66.7 (55.6–77.8)	77.8 (66.7–88.9)	11.1 (0.0–16.7)	<0.001
Respiratory symptoms	66.7 (40.3–77.8)	88.9 (72.2–94.4)	22.2 (11.1–44.4)	<0.001
Digestive symptoms	77.8 (66.7–88.9)	77.8 (66.7–88.9)	0.0 (−19.4 to 11.1)	0.860
Vitality	50.0 (37.5–66.7)	66.7 (50.0–83.3)	8.3 (0.0–25.0)	<0.001
Health perceptions	55.6 (33.3–77.8)	66.7 (55.6–88.9)	11.1 (0.0–22.2)	<0.001
Weight	100.0 (66.7–100.0)	100.0 (66.7–100.0)	0.0 (0.0–33.3)	0.825
Role functioning	83.3 (66.7–91.7)	91.7 (75.0–100.0)	8.3 (0.0–16.7)	<0.001

**TABLE 3:** Sub group analysis of the effects of elexacaftor/tezacaftor/ivacaftor (ETI) in female and male patients with CF on quality of life determined by the Cystic Fibrosis Questionnaire-Revised (CFQ-R), symptoms of depression determined by Patient Health Questionnaire-9 (PHQ-9) and Beck’s Depression Inventory-FastScreen (BDI-FS), and symptoms of anxiety with the Generalized Anxiety Disorder Scale-7 Items (GAD-7).

				
	Female		Male	
Questionaire	Change between baseline and ETI median (IQR)	*p*-Value	Change between baseline and ETI median (IQR)	*p*-Value
CFQ-R respiratory domain	22.2 (IQR 11.1–38.9)	<0.001	22.2 (IQR 6.9–52.8)	<0.001
PHQ-9	0.0 (IQR -2.0 to 1.0)	0.608	−1.5 (IQR -4.0 to 0.0)	<0.001
BDI-FS	0.0 (IQR -0.8 to 0.0)	0.112	0.0 (IQR -1.0 to 0.0)	<0.05
GAD-7	0.0 (IQR -1.0 to 1.0)	0.704	−1.0 (IQR -3.0 to 0.0)	<0.05

### 3.3 Depression

**FIGURE 3:**
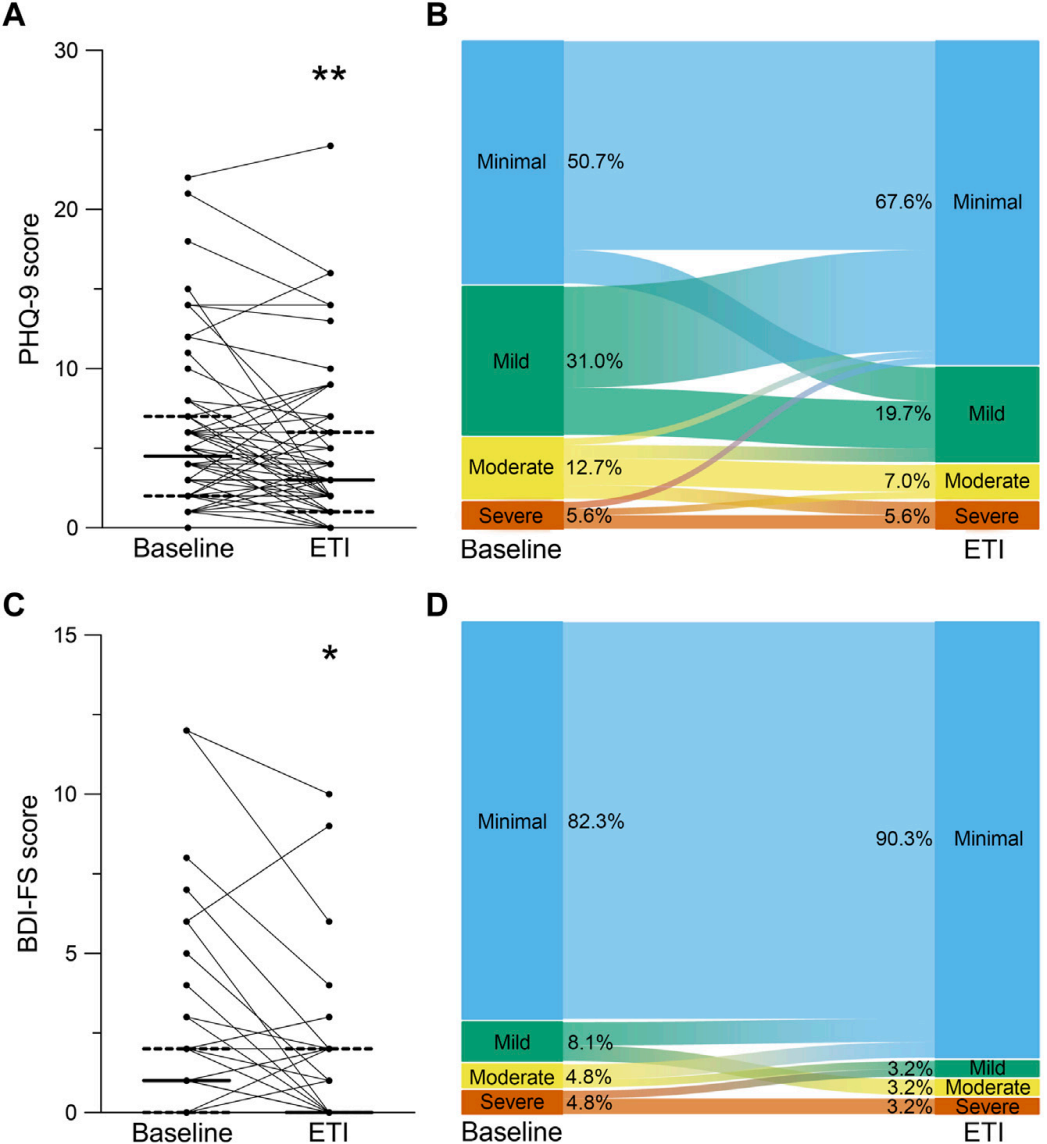
Effects of elexacaftor/tezacaftor/ivacaftor (ETI) on symptoms of depression. **(A–D)** Paired assessment of Patient Health Questionnaire-9 (PHQ-9) **(A,B)** and Beck’s Depression Inventory-FastScreen (BDI-FS) **(C,D)** in patients with CF and at least one *F508del* mutation at baseline and after initiation of ETI therapy. **(B,D)** Alluvial graphic depicting the proportions of the categories minimal (blue), mild (green), moderate (yellow) and severe (red) symptoms of depression assessed by PHQ-9 **(B)** and BDI-FS **(D)** in CF patients at baseline and after initiation of ETI therapy. Solid lines represent the group median and dashed lines represent 25th and 75th percentile. *p < 0.05 and **p < 0.01 compared with baseline.

At baseline, 81.7% reported minimal or mild and 18.3% reported moderate or severe symptoms of depression with a median score of 4.5 (IQR 2.0–6.8) assessed by the PHQ-9 (Figures 3A, B). In the BDI-FS questionnaire, 90.4% of patients reported minimal or mild and 9.6% reported moderate or severe symptoms of depression with a median score of 1.0 (IQR 0.0–2.0) at baseline (Figures 3C, D). After initiation of ETI, PHQ-9 scores decreased by 1.0 (IQR -3.0 to 0.3; *p* < 0.05; Figure 3A). We observed a decrease in mild (−11.3%) and moderate (−5.7%) scores, and an increase in the minimal scores (+16.9%) after initiation of ETI compared to baseline (Figure 3B). The proportion of severe scores did not change after initiation of ETI. BDI-FS scores decreased to 0.0 (IQR 0.0 to 2.0; *p* < 0.05) after initiation of ETI (Figure 3C). Mild (−4.9%), moderate (−1.6%) and severe (−1.6%) scores decreased and minimal scores increased by 8.0% after initiation of ETI (Figure 3D). Further, there was also trend towards a decrease in number of patients describing suicidal ideation. At baseline, 4 patients (5.6%) reported suicidal ideation whereas after initiation of ETI only one patient (1.4%) still reported suicidal ideation. In a gender-based subgroup analysis, both depression scores, the PHQ-9 (−1.5, IQR -4.0 to 0.0; *p* < 0.001) and the (0.0, IQR -1.0 to 0.0; *p* < 0.05) BDI-FS score improved in male patients with CF (Table 3). However, in the female subgroup, no improvement in PHQ-9 (0.0, IQR -2.0 to 1.0; *p* = 0.608) and BDI-FS (0.0, IQR -0.8 to 0.0; *p* = 0.112) were observed after initiation of ETI (Table 3).

### 3.4 Anxiety

**FIGURE 4:**
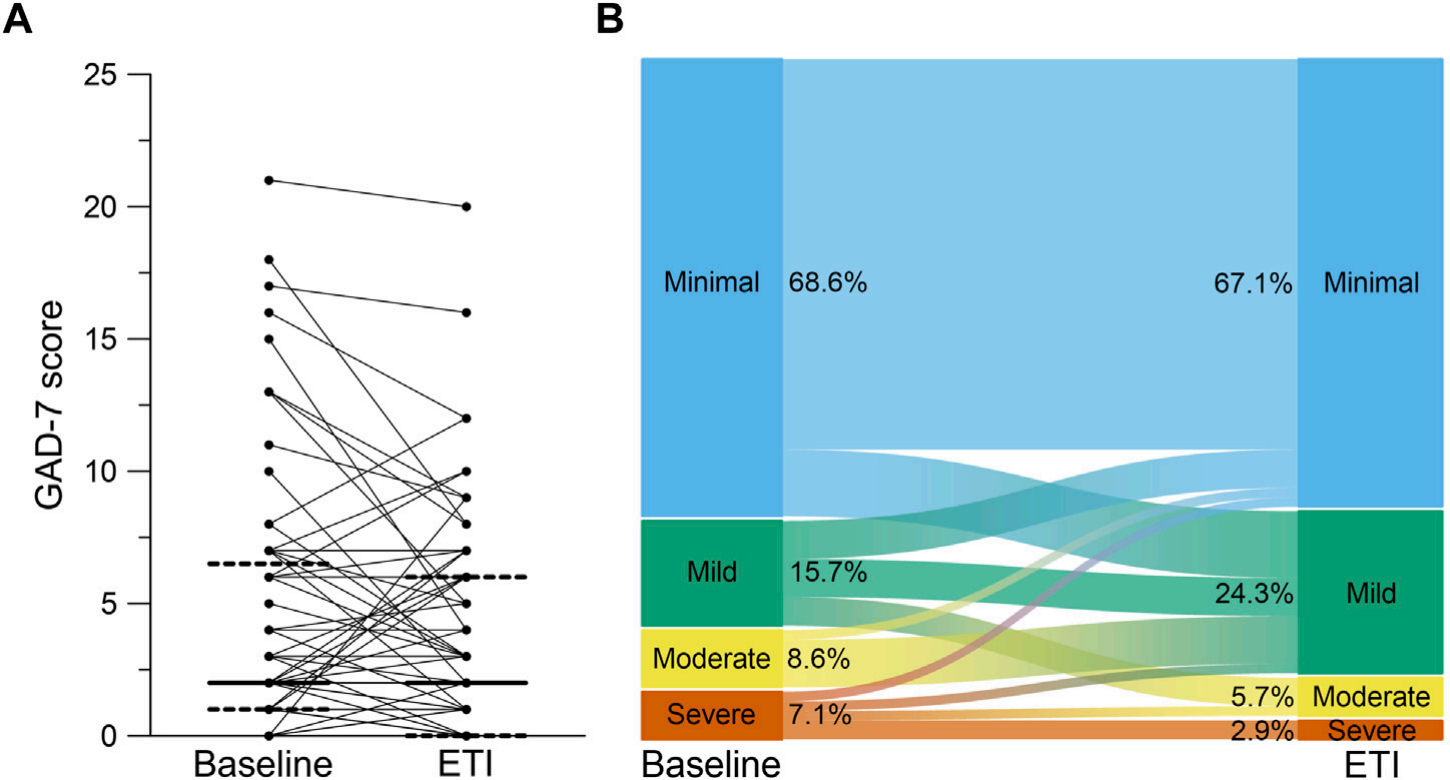
Effects of elexacaftor/tezacaftor/ivacaftor (ETI) on symptoms of anxiety. **(A)** Paired assessment of Generalized Anxiety Disorder Scale-7 Items (GAD-7) in patients with CF and at least one *F508del* mutation at baseline and after initiation of ETI therapy. **(B)** Alluvial graphic depicting the proportions of the categories minimal (blue), mild (green), moderate (yellow) and severe (red) symptoms of anxiety assessed by GAD-7 in CF patients at baseline and after initiation of ETI therapy. Solid lines represent the group median and dashed lines represent 25th and 75th percentile.

At baseline, 84.3% of the patients reported minimal or mild and 15.7% reported moderate or severe symptoms of anxiety with a medium score of 2.0 (IQR 1.0 – 6.0) in the GAD-7 (Figure 4A). After initiation of ETI, GAD-7 scores did not change compared to baseline (median difference 0.0; IQR -2.0 – 0.0; *p* = 0.112; Figure 4A). We observed a trend towards a decrease in the categories of minimal (−1.5%), moderate (−2.9%) and severe (−4.2%) scores and a trend towards increase in mild (8.6%) scores (Figure 4B). In the gender-based subgroup analysis, the GAD-7 scores improved in the male subgroup (−1.0, IQR -3.0 to 0.0; *p* < 0.05), but no change was observed in the female subgroup (0.0, IQR -1.0 to 1.0; *p* = 0.704) (Table 3).

## 4 Discussion

This is the first prospective study assessing the impact of ETI treatment on mental health of patients with CF by using the PHQ-9, BDI-FS and GAD-7 questionnaires in a post-approval, real-world setting. In a cohort of 70 adult patients with a broad range of disease severity, the improvements in key clinical outcomes ppFEV1 and BMI, as well as sweat chloride concentration and quality of life, observed after initiation of ETI therapy were consistent with the results obtained in phase three clinical trials and previous observational studies in real-life settings (Tables 1, 2; Figure 2) (Heijerman et al., 2019; Middleton et al., 2019; Barry et al., 2021; Burgel et al., 2021; Graeber et al., 2022a; Nichols et al., 2022). We found that ETI therapy improves symptoms of depression in patients with CF with at least one *F508del* allele (Figure 3). Further, we show that ETI does not alter symptoms of anxiety in the whole cohort (Figure 4). Subgroup analysis showed that symptoms of depression and anxiety were reduced in male but not in female patients (Table 3). Collectively, our results provide novel insights into the short-term treatment with ETI on symptoms of depression and anxiety in adult patients with CF.

The quality of life assessed by the CFQ-R showed improvement in most domains after initiation of ETI in our study (Table 2). However, the emotional functioning domain and domains associated with eating and digestion, such as eating problems, digestive symptoms and weight, showed no improvement (Table 2). An analysis of the non-respiratory health-related quality of life during the previous phase 3 clinical trials showed that all, but the digestive symptoms domain were improved after initiation of ETI (Fajac et al., 2023). However, similar to our study, another prospective real-world study observed no improvement for the emotional functioning, health perceptions, body image, and digestive symptom domains (DiMango et al., 2021). The emotional function items in the CFQ-R also partially assess symptoms of anxiety which, in line with the results of the GAD-7, could potentially explain why this domain was not improved.

In our study, we observed slightly fewer symptoms of depression and anxiety compared to previous studies on the prevalence of mental health issues in patients with CF in Europe (Figures 3, 4) (Goldbeck et al., 2010; Yohannes et al., 2012; Quittner et al., 2014; Graziano et al., 2020). This observation may be explained by the positive reports on ETI accompanying the approval as breakthrough therapy by the U.S. Food and Drug Agency in October 2019 and, therefore, the anticipation of starting with a highly effective CFTR modulator therapy at the baseline visit.

Recently, some case reports suggested potential side effects of ETI on mental health with an increase in depressive symptoms and even suicide attempts in patients with CF (Tindell et al., 2020; Ladores and Polen, 2021; Heo et al., 2022; Arslan et al., 2023). One study suggested a dose reduction in individuals with reported mental health issues after initiation of ETI, which resulted in improvement or resolution of symptoms of depression and anxiety (Spoletini et al., 2022). However, a retrospective analysis observed no significant changes in average PHQ-9 and GAD-7 scores after initiation of ETI (Zhang et al., 2022). In contrast, in our prospective study, we observed an improvement in depressive symptoms in PHQ-9 as well as BDI-FS scores after short-term treatment with ETI (Figure 3). A potential mechanism was reported in a mouse model of depression suggesting potentially beneficial effects of ivacaftor and its metabolites on the central nervous system activity profile (Schneider et al., 2018). Further, sleep quality improved in 50% of patients with CF and advanced lung disease after initiation of ETI, which could contribute to the improvement in depressive symptoms (Martin et al., 2021). Overall, two patients changed from moderate to severe depressive symptoms in the PHQ-9 in our study (Figure 3). However, both patients reported other potential causes for worsening of symptoms (problems at work and separation of partner) besides initiation of ETI underlining the multiple factors influencing mental health.

Symptoms of anxiety did not change after initiation of ETI therapy (Figure 4) which is in line with a previous retrospective study (Zhang et al., 2022). In contrast to previous case reports (Tindell et al., 2020; Spoletini et al., 2022), we did not observe an increase in anxiety symptoms on the group level. Further, only three patients changed from mild to moderate symptoms of anxiety and no patient changed to severe symptoms of anxiety after initiation of ETI (Figure 4). Another case report suggests increased symptoms of anxiety due to the life-changing effects of ETI including the fear of diminishing effectiveness over time (Ladores and Polen, 2021). However, further studies in larger patient populations will be necessary for a more comprehensive elucidation of the effects of ETI on mental health in patients with CF.

Subgroup analysis showed that although baseline values in females and males were comparable, symptoms of depression and anxiety improved in male but not in female patients with CF (Table 3). Recent case reports indicate a higher likelihood of mental health issues in females compared to males after initiation of CFTR modulators (Tindell et al., 2020; Ladores and Polen, 2021). Female sex is further associated with lower survival rates, earlier bacterial colonization, higher decrease in lung function and more frequent pulmonary exacerbations in patients with CF (Harness-Brumley et al., 2014; Montemayor et al., 2021; Montemayor and Jain, 2022; Sodhi et al., 2023). However, there was no difference in the effects on the CFQ-R respiratory domain score and lung function between female and male patients highlighting that the effects of ETI on mental health may not be directly attributable to clinical improvements. This highlights that the underlying mechanisms of the sex differences are still unknown. Further, it is possible that the sample size in our study was not sufficient to detect more subtle effects in female patients. Therefore, larger studies powered for gender-specific differences are necessary to further elucidate this finding.

This study has some limitations. As the approval of ETI in Europe took place during the COVID-19 pandemic, this might have influenced our study results (Sakon et al., 2023). However, we observed an improvement of symptoms of depression despite the ongoing COVID-19 pandemic. The missing effects of ETI on symptoms of anxiety could be especially influenced by the pandemic as anxiety scores were observed to be elevated in the general population during the COVID-19 pandemic (Salari et al., 2020), potentially resulting in an overlap with a possible reduction of anxiety following ETI. Second, the questionnaires used for anxiety and depression are self-report measures that are useful for screening of depressive and anxiety symptoms but may lack sensitivity and might therefore not capture the full range of symptoms or severity. Novel, more CF specific questionnaires, like the recently developed Distress in Cystic Fibrosis Scale (DCFS) may provide a more comprehensive assessment of the mental health of patients with CF (Finlay et al., 2022). Third, no neuropsychiatric and neurocognitive symptoms that were recently described to be altered after initiation of ETI (Aspinall et al., 2022; Spoletini et al., 2022), nor social characteristics such as education, working and relationship status were assessed in this study. Further, this prospective real-world study with a limited sample size assessed only short-term effects of ETI. However, in a case series on patients with changes in mental health, all six patients noticed a change within the first 3 months after initiation of ETI therapy (Heo et al., 2022) that are covered in our cohort. Nevertheless, larger, multi-center, longitudinal studies over longer time periods will be necessary to identify potential long term effects of ETI on mental health.

In summary, our study demonstrates that initiation of ETI therapy leads to improvement in symptoms of depression and does not change symptoms of anxiety on a group level in adult patients with CF. However, as multiple factors influence mental health, we suggest that mental health screening including neurocognitive and neuropsychiatric symptoms should be routinely performed also after initiation of ETI in all patients with CF to identify individual patients with an increase of symptoms of depression and anxiety.

## Data Availability

The raw data supporting the conclusions of this article will be made available by the authors, without undue reservation.
